# The Pandemic Effect: Secondary Impact on the Diagnosis of Clinically Significant Congenital Heart Disease and Role of Deprivation Index

**DOI:** 10.1007/s00246-025-03844-2

**Published:** 2025-04-03

**Authors:** Cassandra Campbell, Kristin Wyckoff, Ronak Naik, Nithya Swaminathan, Salima Ahmed Bhimani, Jason Johnson, Vijaya Joshi, Ranjit Philip

**Affiliations:** 1 The Heart Institute at Le Bonheur Children’s Hospital and the University of Tennessee Health and Science Center, 51 N. Dunlap, Memphis, TN 38103 USA; 2 University of Tennessee Health Science Center, Memphis, TN USA; 3 The University of Texas Health Science Center at Houston, Houston, TX USA

**Keywords:** Prenatal diagnosis (PND), Prenatal care (PNC), COVID, Social deprivation index (SDI), Congenital heart disease (CHD)

## Abstract

We sought to determine the impact of the COVID-19 on prenatal diagnosis (PND) of clinically significant congenital heart disease (CHD) and the role of socioeconomic status (SES), complexity of diagnosis, and proximity to advance testing. This single-center retrospective study evaluated 2 eras of infants (COVID (born July 1, 2020–July 31, 2023) and pre-COVID (born June 1, 2017–July 1, 2020) who had cardiac surgery in the first year of life. 512 infants, 292 in pre-COVID era and 220 in COVID era with no significant difference in the rate of prenatal care (PNC) or PND in the COVID era (88%/42%) versus pre-COVID era (93%/48%) (*χ*^2^ = 3.22, *p* = 0.07, *χ*^2^ = 1.9, *p* = 0.17). Distance from advanced testing had no influence on PND in the COVID era [55% close versus 53% further away (*χ*^2^ = 2, *p* = 0.65)]. When evaluating SES with income per zip code, the higher SES group had increased PND during the pandemic compared to both pre-COVID era and low SES group. However, social deprivation index (SDI) based on zip code showed the higher SES group had a decrease in PND rates. Both metrics showed no change in PND in the lower SES group during COVID. COVID-19 had no significant change in the PND of clinically significant CHD during the pandemic. The differing SES results using income versus SDI of patient zip codes suggest that barriers to PND is multifactorial. The discrepancy in PND reflects poor referral rates to advanced testing, highlighting the importance of educating frontline healthcare professionals to improve outcomes.

## Background

Prevalence of congenital heart disease (CHD) in North America is 6.9 per 1000 live births [1]. Clinically significant heart defects are those that require intervention in infancy to prevent death with critical CHD requiring intervention within the first week of life. A study from Netherlands showed that the prevalence of clinically significant and critical CHD needing cardiac surgery in the first year of life is 4 in 1000 live births [2]. Prenatal diagnosis (PND) of clinically significant or critical CHD allows for timely multidisciplinary care. This includes specialized prenatal and antenatal management of critical CHDs which is required in the delivery room or immediately after birth [3]. PND also allows for education and counseling about CHD, anticipated management, and intervention options with the family. Studies have shown a reduction in mortality and morbidity with prenatal detection of critical CHD such as hypoplastic left heart syndrome, coarctation of the aorta, and D- transposition of the great arteries [2]. However, based on a 2015 study by Quartermain et al. only about 42% of babies with critical CHD in the United States had a diagnosis before birth with variability based on geographic regions [4].

The current recommendation for cardiac assessment is at 18–22 weeks of gestation [5]. This is when a mid-gestational ultrasound is performed for fetal anomalies. If CHD is detected then the patient should be referred to a center capable of performing fetal echocardiogram (i.e., advanced testing) for more detailed assessment [6]. During the COVID-19 pandemic, there were significant strains on the healthcare system that at times created barriers to patient care [7]. Resources were limited and had to be re-allocated. Staffing was low due to illness or reassignment to areas of need in the hospital intensive care units and wards. Extra precautions to keep patients and staff safe specifically in the sphere of echocardiography and general ultrasound included modifications in practice such as a relative lesser frequency of appointments to reduce traffic and accommodate time for cleaning equipment in between studies [8]. There were also barriers on the patient's side such as loss of health insurance due to job loss and fear of contracting COVID while at a medical facility. It has been shown that these barriers reduced access to patient care and that those with low socioeconomic status (SES) and low health literacy were affected the most [9]. Families with lower SES have been associated with lower rates of prenatal detection of CHD [10].

Considering all these factors, we hypothesized that pandemic related health barriers decreased the rate of PND of major CHD and worsened any existing barriers that SES had created. The primary aim was to determine if there was a reduction in PNC or PND of clinically significant CHD during the COVID-19 pandemic in our region. The secondary aim was to evaluate if the barriers created by the pandemic compounded existing factors such as SES, diagnosis complexity, or distance from advanced testing centers.

## Methods

This is a single-center retrospective study of infants born between 6/1/2017 and 7/31/2023 who had cardiac surgery for clinically significant CHD which was defined as requiring surgery in the first year of life [2]. Infants excluded were those referred through the gift of life program, cardiomyopathy, cardiac tumors, and surgical patent ductus arteriosus (PDA) closure. Gift of Life patients were excluded due to not having PNC in the United States. Cardiomyopathy and cardiac tumors were excluded as these may not have been present prenatally and hence if not diagnosed during PNC, they were not necessarily a “missed” PND. Surgical PDA closures were excluded as a PDA is a normal fetal finding. The infants were divided into COVID and pre-COVID eras for comparison. The COVID era was defined as patients born between July 1, 2020 and July 31, 2023. These dates were chosen because patients would have been less than 20 weeks gestation during the pandemic (March 11, 2020 to May 11, 2023) as declared by the World Health Organization [11]. Those who reached 20 weeks of gestation during that time-frame, potentially had decreased access to their anatomy scan and were more likely to have a missed diagnosis. The pre-COVID cohort was the equivalent amount of time, i.e., the 3-year period (June 1, 2017 to July 1, 2020) prior to the pandemic. Metrics of comparison between groups were prenatal care (PNC), PND, surgical complexity of CHD, distance to advanced testing, and SES.

### Prenatal Care and Prenatal Diagnosis

The determination of having received PNC was reliant on the parent’s self-reporting of having an obstetrician that they followed with regularly during their pregnancy. A CHD diagnosis prior to birth was the criteria for having a PND.

### Surgical Complexity of Congenital Heart Disease

Complexity was defined using The Society of Thoracic Surgeons—European Association for Cardio-Thoracic Surgery Congenital Heart Surgery Mortality Categories (STAT score) which was created to have an objective index of risk of mortality of cardiac surgery by procedure [12]. Procedures associated with lowest mortality rates are in Category 1 and procedures associated with highest mortality rates are in Category 5. Examples are seen in Table 1.

**Table 1:** Examples of congenital heart operations in each STAT category

STAT Score	Congenital heart operation
1	Patch repair atrial septal defect, ventricular septal defect, pulmonic valve replacement, vascular ring
2	Ross procedure, Fontan procedure, tricuspid valve repair, coarctation, Glenn, coarctation, Tetralogy of Fallot
3	Complete atrioventricular canal repair, Arterial Switch Operation, Rastelli
4	Blalock–Taussig–Thomas shunt, double outlet right ventricle, Truncus arteriosus repair, total anomalous pulmonary venous return
5	Heart and lung transplant, Norwood procedure, truncus plus interrupted arch repair

### Referral and Distance to Advanced Testing

Referral to advanced testing was defined as the patient having a fetal echocardiogram performed by a fetal cardiologist associated with a tertiary care center. The distance to advanced testing was based on the patient zip code distance from the tertiary care zip code. Patients were considered close to advanced testing if their zip code was within the same county as the tertiary care center, approximately a 30 min drive. Patients were considered far away if their zip code was outside this perimeter, approximately > 30 min drive.

### Socioeconomic Status

The patient’s zip code was also used to determine SES. Zip codes were used as opposed to obtaining individual incomes because one of the secondary aims was to identify regions where more targeted ultrasound training would be beneficial. Mean annual income of each zip code was obtained from the US Census bureau for 2019 [13]. Lower SES was defined as a zip code with mean income less than or equal to Medicaid qualification level for a family of 3 in 2019, $33,064. Higher SES was defined as a zip code with mean income greater than Medicaid qualification level for a family of 3 in 2019, $33,064 [14]. Additionally, we used the SDI to identify SES as an additional metric for assessment of barriers to healthcare. This index was created by the Graham Center in association with the American Academy of Family Physicians to quantify levels of disadvantage across in the United States and evaluate their associations with health outcomes to better address health inequities [15]. This tool uses 7 categories from the American Community Survey to then quantify socio-economic level based on zip code. The 7 categories include the percentage of people living in poverty, with less than 12 years of education, single-parent households, living in rented housing units, living in the overcrowded housing unit, households without a car and the percentage of non-employed adults under 65 years of age [16]. We categorized the patients into low and high SDI groups based on a cut off of 58 where above 58 is a high deprivation index and below 58 is a low deprivation index and therefore higher SES [15].

### Data Analysis

The study involved retrospective review of the electronic medical record. The University of Tennessee Institutional Review Board waived the need for parental consent and approved this study. Data analysis was performed utilizing the SPSS software Version 25 for all statistical analysis (SPSSS, Chicago, IL). All metrics are compared as categorical variables. Categorical variables are expressed as numbers and percentages. The variables were analyzed using chi square with a *p* value < 0.05 deemed statistically significant.

## Results

**Fig. 1:**
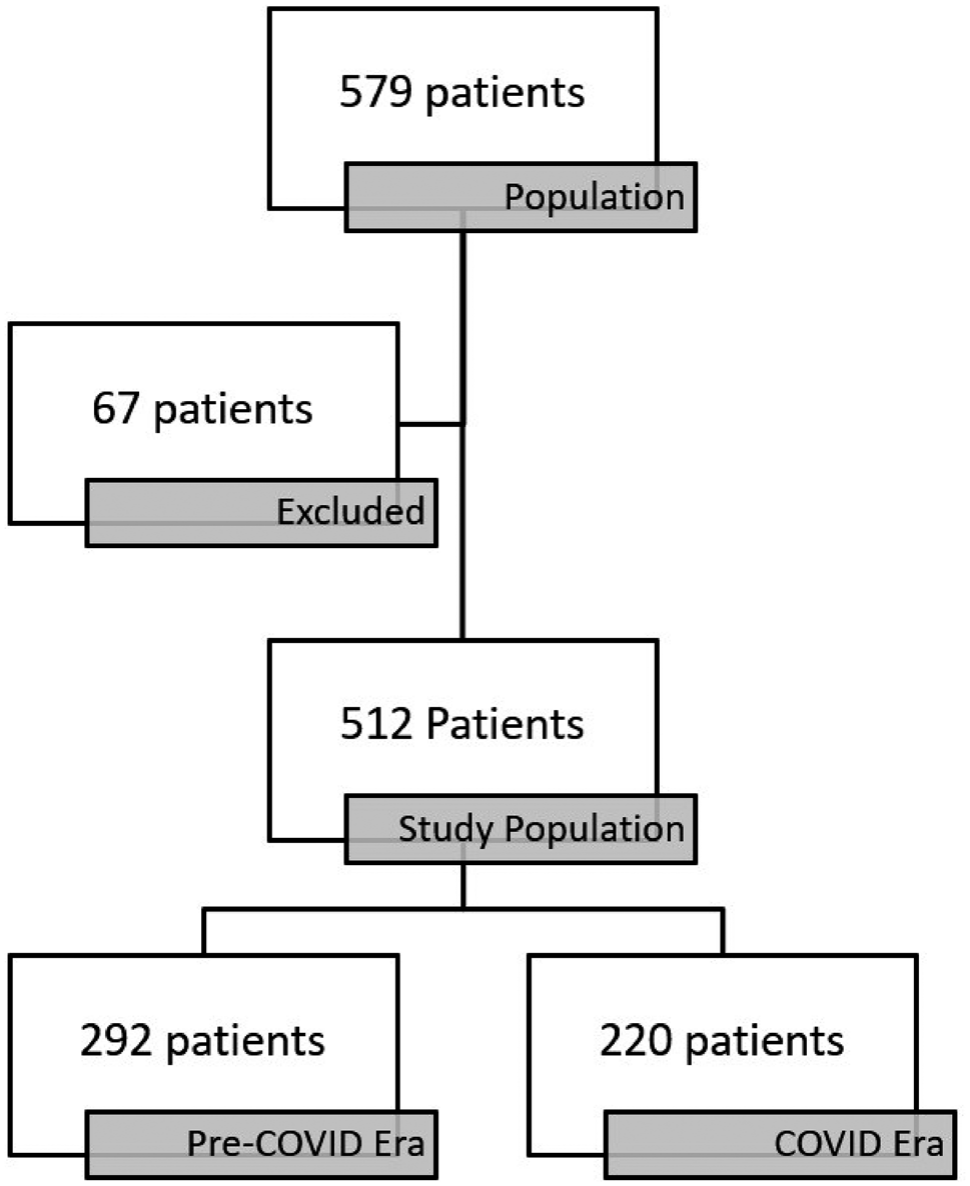
Patient population: relative decrease in the number of major cardiac surgeries during the COVID era (March 11, 2020 to May 11, 2023) compared to the equivalent time-frame in the pre-COVID era. Infants excluded were those referred through the gift of life program, cardiomyopathy, cardiac tumors, and surgical patent ductus arteriosus (PDA) closure

A total of 579 patients were enrolled in the study, 67 were excluded leaving a total of 512 patients. 292 patients were in the pre-COVID era and 220 patients were in the COVID era (Fig. 1).

### Prenatal Care and Prenatal Diagnosis

There was no significant difference in the rate of PNC in the COVID era when compared to the pre-COVID era with 88% of parents reporting PNC during the COVID era versus 93% of parents reporting prenatal care prior to the COVID-19 pandemic (*χ*^2^ = 3.22, *p* = 0.07) (Table 2). Similarly, there was no significant difference in rate of PND in the COVID era when compared to the pre-COVID era with 42% of patients having a CHD diagnosis before birth during the COVID era versus 48% during the pandemic (*χ*^2^ = 1.9, *p* = 0.17) (Table 2).

**Table 2:** Comparison of patients that had PNC in the pre-COVID and COVID eras and comparison of patients that had a PND in the pre-COVID and COVID eras

	Pre-COVID	COVID	Total	
Prenatal care (PNC)				
Yes	271 (93%)	194 (88%)	465	*χ*^2^ = 3.22, *p* = 0.07
No	21 (7%)	26 (12%)	47	
Total	292	220	512	
Prenatal diagnosis (PND)				
Yes	140 (48%)	92 (42%)	232	*χ*^2^ = 1.9, *p* = 0.17
No	152 (52%)	128 (58%)	280	
Total	292	220	512	

### Surgical Complexity of Congenital Heart Disease

**Fig. 2:**
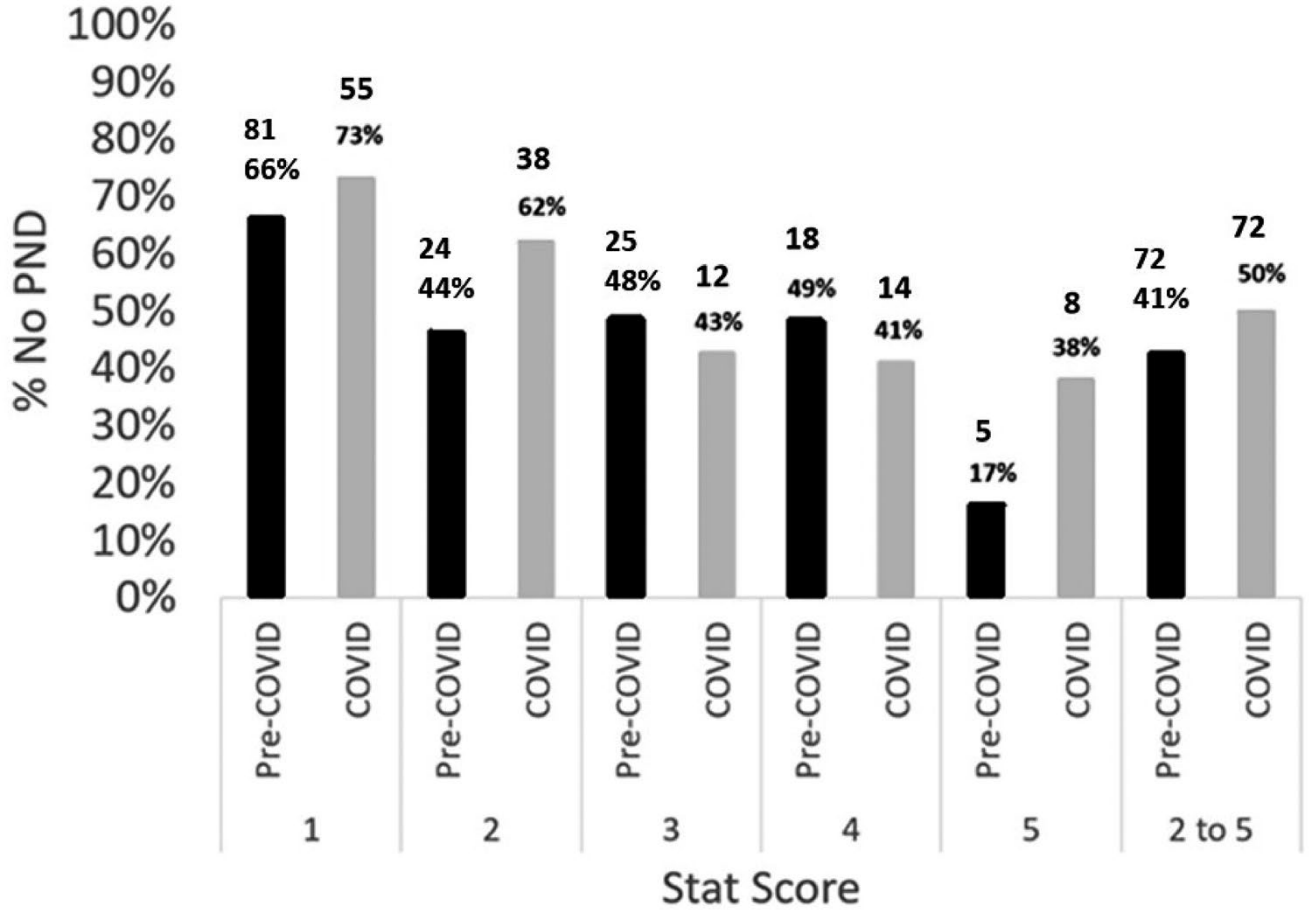
Percentage and number of patients with no PND in the pre-COVID and COVID eras for each STAT score 1 to 5 and percentage of patients with no PND in the pre-COVID and COVID eras for all STAT scores 2 to 5

When stratified by surgical STAT score, where STAT 1 category includes the least complex operations and STAT 5 category includes the most complex operations, there was no significant difference in PND rates between the two eras (Fig. 2). The rate of PND in the COVID era (38%) for infants that underwent a STAT 5 surgery was not statistically significant (*χ*^2^ = 2.99, *p* = 0.084) in comparison to the pre-COVID era (17%). As expected, the lower complexity lesions (STAT 1 group), had a much higher rate of missed PND regardless of era [(66% pre-COVID versus 73% during the COVID era (*χ*^2^ = 2.99, *p* = 0.084)]. Even when the STAT 1 group was excluded, the rate of missed PND was greater than 40% when averaged across STAT scores 2 to 5 and not significantly different between pre-COVID and COVID eras [41% versus 50% during the COVID era (*χ*^2^ = 0.71, *p* = 0.19)] (Fig. 2).

### Referral and Distance to Advanced Testing

When comparing patients close to a tertiary care center versus those further away, living within 30 min of the tertiary care center did not increase the number of patients with a PND during the COVID pandemic. 14 of 40 (35%) whose parents resided in Shelby County, TN had a prenatal diagnosis compared with 79 of 180 (44%) whose parents resided outside of Shelby County, TN did not have a prenatal diagnosis (*X*^2^ = 1.0697; *p* = 0.3033). There was no difference in the prevalence of PNC between the COVID and pre-COVID eras after adjusting for residence close to the tertiary care center (OR 0.815; 95% Confidence Interval 0.572, 1.161). There was also no difference in the prevalence of PNC between those living close to the tertiary care center versus far away after controlling for the era (OR 1.367; 95% Confidence Interval 0.867, 2.157). Overall, almost all patients were within a 120-mile radius of the tertiary care center.

### Socioeconomic Status

When evaluating the effects of SES based on income by zip codes, there was available income data on zip codes for 420 of the 512 infants. The cohort of infants with lower SES living in zip codes with mean annual income less than or equal to Medicaid qualification level for a family of 3 were 22% (*n* = 93) of the 420 that were evaluated. In this cohort, there was no significant difference in PND regardless of the era [36% COVID era versus 41% pre-COVID era (*χ*^2^ = 0.224, *p* = 0.63)]. However, for those with higher SES living above the annual income for Medicaid qualification level for a family of 3 there was a significant increase in the rate of PND in the COVID era compared to pre-COVID era. [75% COVID era versus 45% pre-COVID era (*χ*^2^ = 26.9, *p* < 0.0001)]. The higher SES group also had a significant increase in PND during the COVID era compared to lower SES group [75% high SES versus 36% low SES (*χ*^2^ = 20.02, *p* < 0.0001)]. (Table 3). The relationship between SES and PND was also evaluated using SDI. This takes into account more than income when evaluating deprivation.

**Table 3:** Comparison of patients that lived in a zip code where the mean annual income was less than or equal the Medicaid qualification level for a family of 3 in 2019 ($33,064) in the pre-COVID and COVID eras

	Pre-COVID	COVID	Total	
Mean annual income ≤ Medicaid qualification level for a family of 3 ($33,064)				
PND	22 (41%)	14 (36%)	36	*χ*^2^ = 0.224, *p* = 0.63
No PND	32 (59%)	25 (64%)	57	
Total	54	39	93	
Mean annual income > Medicaid qualification level for a family of 3 ($33,064)				
PND	101 (45%)	79 (75%)	180	*χ*^2^ = 26.9, *p* < 0.0001
No PND	122 (55%)	25 (25%)	147	
Total	223	104	327	

**Fig. 3:**
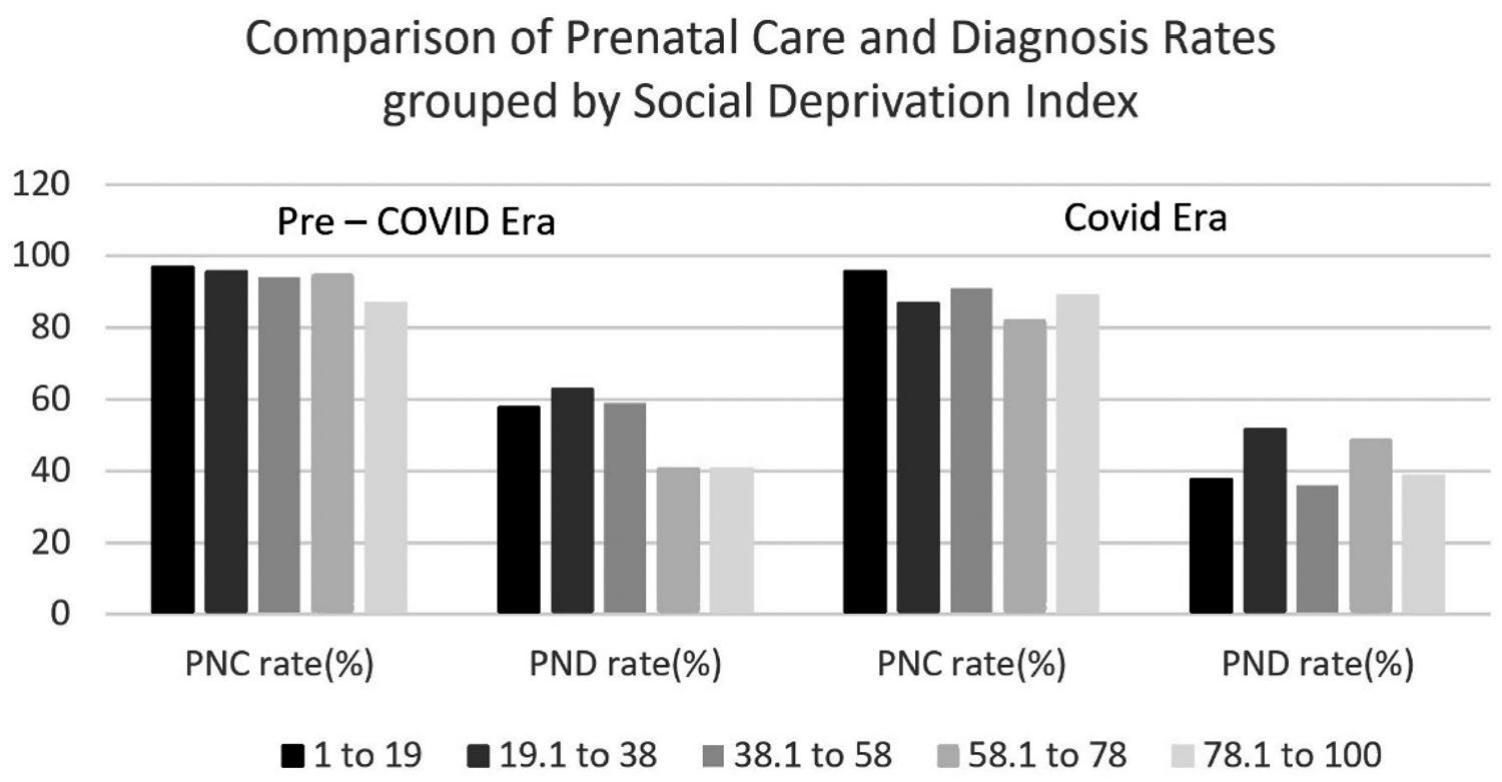
Percentage of PNC and PND in each SDI quartile in the pre-COVID and COVID eras

The relationship between the SDI score and the severity of deprivation is direct, positive, and linear; as the SDI score increases, the severity of deprivation increases too. In the pre-COVID era, the PNC rates were high in both groups (96% versus 91% for low and high SDI, respectively). However, there was a significantly higher chance of no PND among those who received PNC in the higher SDI group, i.e., lower SES (51% versus 38%, Odds ratio 1.68, 95% CI 1.0–2.8, *p* = 0.024). During the COVID era, the PNC rates in the low and high SDI groups were relatively lower than the pre-COVID era at 91% and 86%, respectively. There was a high rate of no PND among those who got PNC in the COVID era but there was no significant difference between the SDI groups (52% in the low SDI group and 49% in the high SDI group). There was a significantly higher rate of no PND in the COVID era in the low SDI group in comparison to the pre-COVID era (52% versus 38%, Odds ratio 1.9, 95% CI 1.1–3.5, *p* = 0.04). However, there was no significant difference in PNC and PND rates in the high SDI group regardless of the era (91% and 42% pre-COVID versus 86% and 43% in COVID era, *χ*^2^ = 0.15, *p* = 0.69) (Fig. 3).

When SDI was considered in the model as a continuous variable, there was no difference in prevalence of PNC between the COVID years compared to the pre-COVID years (OR 0.800; 95% Confidence Interval 0.560, 1.143). When SDI was stratified into five groups of increasing deprivation and included in the model, SDI was not a significant predictor of PND (OR 1.133; 95% Confidence Interval 0.997, 1.288). Overall, there was higher deprivation index, thus low SES, in patients with no PND in comparison to patients with a PND (65.47 versus 60.24, Two sample *T* test =  −  2.01, *p* = 0.045) this was also true pre-COVID (67.74 versus 59.54, Two sample *T* test = − 2.44, *p* = 0.0151). However, during COVID there was no significant difference in deprivation index between no PND and PND (62.74 versus 61.27, Two sample *T* test = − 0.359, *p* = 0.719) (Table 4).

**Table 4:** Two sample *T* test comparing average SDI in patients with no PND versus PND

	No PND	PND	Two sample *T* test
All patients	65.47	60.24	− 2.01, *p* = 0.045
Pre-COVID	67.74	59.54	− 2.44, *p* = 0.0151
COVID	62.74	61.27	− 0.359, *p* = 0.719

## Discussion

Contrary to our hypothesis, this study showed that in patients with clinically significant CHD, there was no significant difference in the rates of PNC and PND during the COVID-19 pandemic. The compounding effects of a lower SES during the pandemic did not have a negative effect on these rates when using SDI as a metric. One might argue that there are negative consequences of known PND including increased maternal stress and anxiety therefore why should one worry about improving rates of prenatal diagnosis. Pregnant mothers have more frequent doctor appointments and parents have the psychological stress of preparing for an ill child [3]. There is a perception that the innate known effects of the pandemic on mental health [17] may possibly have further compounded the maternal stress associated with a PND. There are also unclear impacts of PND on the morbidity and mortality of CHD. However, studies have shown a reduction in mortality with critical CHD which is why we feel it is important to improve PND [2].

The reduction in cardiac surgeries during the pandemic seen in our cohort was also seen worldwide. This has been attributed to the decrease in elective surgeries as a consequence of decreased ECMO availability, decreased staff, and decreased ICU bed availability [18]. However, it may also reflect the steady reduction in the fertility rate in the United States over the last decade [19]. Internationally, some countries had a decline in birth rate due to an increase in stillborn rate during the pandemic and women who contracted COVID during pregnancy were more likely to miscarry [20]. Because our study only looked at patients that underwent surgery, it did not account for patients with CHD that died before surgery. This could have affected our rates of PND. However, based on the CDC’s fetal mortality statistics the overall downtrend of fetal mortality in US that began before the 1990s continued during the pandemic with no significant change between 2020 and 2021 [21].

Obstacles in access to healthcare during the pandemic were postulated reasons to find a decrease in rates of PNC and PND in infants with clinically significant CHD. Both, healthcare facility factors such as decrease in staffing and resources as well as patient factors such as loss of healthcare coverage and the fear of getting sick were hurdles to access [9]. However, this study revealed that there was no decrease in PNC or PND during the pandemic. This is potentially due to the rise in telemedicine with virtual consultations replacing in person visits, the increased use of home monitoring devices, and enhanced public health messaging during the pandemic that may have contributed to stabilizing the PND rate. These results are similar to the CDC’s National Vital statistics has shown that during the pandemic there was only a slight increase in mothers with no prenatal care from 1.9 to 2.1% and overall women presented earlier in pregnancy (< 4 months gestation), but had less prenatal visits compared to pre-pandemic [22]. Since it can be hard to visualize the heart adequately later in pregnancy, one might argue that if women were coming earlier in gestation there would have been a higher PND rate of clinically significant CHD, however some lesions are progressive and not evident until later in gestation.

There was no difference in the PND based on surgical STAT score as an indicator of complexity when comparing the eras. As expected, due to minimal shunting in utero, the lower complexity lesions like ventricular septal defects and atrial septal defects had a higher rate of missed diagnosis compared to more complex lesions regardless of the era. Nevertheless, even with these excluded, the more complex lesions were not more likely to be diagnosed. This seems in contrast to a single-center study by Gupta et al. where they noted an increase in prenatal diagnosis of lesions involving outflow tract anomalies and single ventricles during the pandemic. These are typically more complex lesions [23]. There are several studies on the rates of prenatal detection of CHD with differing results which highlights regional differences playing a larger role as opposed to national level differences [24]. This was also again evident during the pandemic with a single-center study in California having an increased rate of CHD diagnosis during the pandemic which differs from our mid-south region [23].

There are studies that have suggested the impact of lower SES on the rates of PND [10, 25]. A study looking at fetuses and infants < 2 months of age with hypoplastic left heart syndrome and d-transposition of the great arteries admitted between 2012 and 2016 to participating Fetal Heart Society Research Collaborative institutions in the United States and Canada, showed that lower SES was associated with decreased PND of hypoplastic left heart syndrome and d-transposition of the great arteries [25]. Three single-center studies, one in San Diego County, another study in Wisconsin, and a third at Boston Children’s found that those living in impoverished or rural communities had lower rates of PND [26–28]. In California the discrepancy was only seen in Hispanic communities [27]. Our study had similar findings with higher deprivation index, thus a lower SES, associated with no PND. With this population known to be disproportionately affected in PND rates and COVID-19, we expected the culmination of these factors to further worsen PND rates [29]. When evaluating SES based on income for each patient zip code, the COVID-19 pandemic did not have an effect on the lower SES group with similarly low rates of PND regardless of the era. Based on the same metric, the higher SES group had an increased rate of PND during the pandemic in comparison to the pre-COVID era as well as in comparison to the lower SES group during the pandemic. The possible explanation for this is that there was plausibly better awareness and relatively higher anxiety related to health during the pandemic for the higher SES group in addition to having more resources at their disposal. When using SDI for each patient zip code as a metric for SES, the lower SES group was not affected by the pandemic in terms of PNC and PND. However, the higher SES group (based on a lower SDI) had a relative decrease in PND rates. This was contrary to the findings using income as a metric. The difference seen with using SDI of a zip code, which takes several socioeconomic factors into account, versus mean income of a zip code makes it evident that income alone does not give a complete picture of the barriers to care families face.

A study in Alberta, Canada found that distance from a tertiary care center affected PND with patients living > 100 km away more likely to have a missed diagnosis or diagnosis after 22 weeks [30]. We were expecting similar findings in our study. However, when comparing PND in patients that lived within the catchment area vs outside the catchment area there was no difference in PND suggesting there are other factors playing a role. This is similar to results from a single-center study at Boston Children’s which showed geographic factors such as driving distance to the nearest center not associated with PND of critical CHD, instead SES played a larger role [28]. When specifically looking at fetuses and infants with hypoplastic left heart syndrome or d-transposition of the great arteries participating in the Fetal Heart Society Research Collaborative institutions in the United States and Canada, there was only a decrease in PND when further from a tertiary care center in patients in Canada [25]. This suggests that transportation differences may be an important factor in certain regions.

While it is encouraging that there was no change in PNC and PND of CHD in our region during the pandemic, the overarching concern is that the overall PND rate was low (< 50%) before and during the pandemic especially when compared to ~ 90% of mothers reporting they had PNC. One of the reasons for this discrepancy could be inadequacy of prenatal care (late entry, late or limited ultrasound), which we were not able to tease out given self-reporting of prenatal care and lack of maternal records. An additional explanation is low rates of referral to advanced testing with a maternal fetal medicine specialist or a fetal cardiologist. Unfortunately, the average time for the mid-gestation anatomy scan is about 20 min with about 5 min for the heart [2]. The standard evaluation is a 4-chamber view of the heart which only detects up to 50% of major congenital heart lesions [3]. The 2013 guidelines on obstetrical ultrasound from the American Institute of Ultrasound in Medicine (AIUM), suggested incorporation of imaging of the outflow tracts during the anatomy scan [31]. At centers where the 3-vessel view and outflow tracts are added this increases sensitivity to 90% [3]. The 2023 guidelines mandate the inclusion of the aforementioned views including the 3-vessel and trachea view [5]. Not all ultrasound centers have implemented these new guidelines and this strategy for screening still misses some anomalies which are subtle at this stage of development and become more obvious later in gestation [2]. Even with the 2013 guidelines, not all centers have seen an increase in PND [24]. This provides an opportunity to educate the obstetricians and sonographers in the community to include the four-chamber view, 3-vessel view, 3-vessel and trachea view and outflow tracts to increase sensitivity to 90% [3] and refer if there are any concerns. This highlights contribution of geographical variation and adherence to application of prenatal screening guidelines as a potential area of further scrutiny in maximizing PND.

This study has provided a roadmap for “areas of need” in our catchment area that may benefit from targeted mid-gestation Ultrasound education for referral for CHD. The National Pediatric Cardiology Quality Improvement Collaborative had provided participating cardiac surgical centers and intensive care units tools (in the form of a provider letter template) for informing pre-natal care centers about the postnatal CHD diagnosis [32]. This initiative was borne from the intent to improve the rate of PND by improving awareness among ultrasound centers that perform the mid-gestation ultrasound. Unfortunately, this has not translated to improved PND rates in our region. Going forward we intend to use what we have learned from this study to provide a collaborative approach to training the frontline sonographers in areas of higher rates of missed diagnosis and low referral rates. One of the gains of the pandemic has been the growth of virtual video-conferencing meetings to extend the reach of educational symposiums [33]. This study has provided a framework to impart targeted hands-on training to zip codes with lower PND rates while providing virtual educational opportunities to the general catchment area. This lays the foundation for quality improvement in this realm while optimizing resource utilization.

There are limitations to this study. Due to only including patients that underwent cardiac surgery in the first year of life, this study is limited by not including patients who had intrauterine fetal demise, elective termination, died before undergoing surgery or only had a cardiac catheterization intervention, which likely affected the number of patients with PND or missed PND. Furthermore, the retrospective study design has an inherent recall bias for identification of PNC. The maternal report of adequate PNC does not indicate that it met the American College of Obstetricians and Gynecologists recommendations. This study also notes that there is a difference in outcomes based on which metric is used for SES and there are several metrics that can be used to look at SES which may continue to provide different results. We also used an indirect approach by income per zip code and SDI, but these measures at a population level do not always correlate on a personal level. Potentially, prospective direct family surveys about their SES would have changed how they were categorized in our study but has inherent biases of reporting in itself. In addition, there was a nationwide stimulus provided during the pandemic that may have helped patients get care they would not have received otherwise. We did not survey our population to determine if this was a factor in patient care.

## Conclusion

In our study, there was an overall decrease in cardiac surgeries during the pandemic. Regardless of the era, despite having high PNC rate, the rate of PND for major CHD was low. Access to care and SES may have compounded the challenges during the pandemic but it did not change rate of detection of CHD in our study population. A targeted and collaborative approach to training frontline sonographers in the community and the wider catchment area could potentially improve the lower rate of referral to advanced testing. This in-turn will possibly lead to decreased rates of missed diagnosis of CHD in the prenatal period. Future studies directed toward assessing geographical variation and adherence to current prenatal screening guidelines may have the potential to improve the national rate of PND.

## Data Availability

Data was obtained from retrospective chart review of the patient's electronic medical record at our institution. The data is stored and protected according to the standards set by the Health Insurance Portability and Accountability Act (HIPAA) therefore cannot be shared openly.
